# NMR Confirmation and HPLC Quantification of Javamide-I and Javamide-II in Green Coffee Extract Products Available in the Market

**DOI:** 10.1155/2017/1927983

**Published:** 2017-08-23

**Authors:** Jae B. Park

**Affiliations:** Diet, Genomics, and Immunology Laboratory, BHNRC, ARS, USDA, Bldg. 307C, Rm. 131, Beltsville, MD 20705, USA

## Abstract

Javamide-I/javamide-II are phenolic amides found in coffee. Recent reports suggested that they may contain several biological activities related to human health. Therefore, there is emergent interest about their quantities in coffee-related products. Green coffee extract is a powder extract made of unroasted green coffee beans, available as a dietary supplement. However, there is little information about the amounts of javamide-I/javamide-II in green coffee extract products in the market. Therefore, in this paper, javamide-I/javamide-II were extracted from green coffee extract products and their identifications were confirmed by NMR. After that, the amounts of javamide-I/javamide-II were individually quantified from seven different green coffee extract samples using the HPLC method coupled to an electrochemical detector. The HPLC method provided accurate and reliable measurement of javamide-I/javamide-II with excellent peak resolution and low detection limit. In all seven green coffee extract samples, javamide-II was found to be between 0.28 and 2.96 mg/g, but javamide-I was detected in only five samples in the concentration levels of 0.15–0.52 mg/g, suggesting that green coffee extract products contain different amounts of javamide-I/javamide-II. In summary, javamide-I/javamide-II can be found in green coffee extract products sold in the market, but their amounts are likely to be comparatively different in between green coffee extract brands.

## 1. Introduction

Coffee plants consist of several species such as* Coffea arabica*,* Coffea canephora*,* Coffea liberica*,* Coffea excelsa*, and* Coffea stenophylla* [1, 2]. Particularly,* Coffea arabica* (Arabica coffee) and* Coffea canephora* (Robusta coffee) are two most commercially important coffee species [2]. Green coffee extract is a powder extract made of unroasted green coffee beans with potential health effects [3–7]. Particularly, green coffee extract is often sold as a weight loss supplement, although more thorough studies are needed to determine its effectiveness as a weight loss supplement [7]. In general, green coffee extract can be made of any unroasted green coffee beans, but most of green coffee extract products found in the market are acclaimed to be made of Arabica beans. Javamide-I (*N*-coumaroyltryptophan) and javamide-II (*N*-caffeoyltryptophan) are safflomide-type phenolic amides found in roasted and unroasted coffee beans [3, 8]. Recently, they were reported to contain several biological activities related to human health [8, 9]. Thus, there is a great deal of interest about how much javamide-I/javamide-II can be found in coffee-related products, particularly green coffee extract found in the market. Therefore, in this paper, javamide-I/javamide-II in the seven green coffee extract products purchased from the market were extracted and their identities were confirmed using NMR spectroscopic methods. Then, their amounts in the seven extract products were determined using the HPLC method with excellent and reliable peak resolution and low detection limit.

## 2. Materials and Methods

### 2.1. Materials

Seven green coffee extract products were purchased from online stores. Javamide-I/javamide-II standards were prepared as reported previously [8, 9]. All other reagents and chemicals were purchased from Sigma Chemical Co. (St. Louis, MO).

### 2.2. HPLC Analysis of Javamide-I/Javamide-II Standards

HPLC analysis was performed as described previously with slight modification [8]. Briefly, a 150 mm × 2.1 mm i.d., 4 *μ*m, Nova-Pak C18 (Waters, Milford, MA) was used as the stationary phase to analyze the standards and green coffee samples. The samples were separated using a gradient condition; buffer A (10 mM phosphate, pH 5) for 0–5 min, a linear gradient from buffer A to buffer B (40% acetonitrile) for 5–45 min, and buffer B for 10 min (1 mL/min). The samples were injected by an autosampler into Alliance 2690 HPLC system (Waters, Milford, MA), and were monitored by CoulArray electrochemical detector with four electrode channels (ESA, Chelmsford, MA).

### 2.3. Extraction of Green Coffee Extract Samples

Green coffee extract samples were extracted in two different ways: collectively (for the NMR analysis and extraction recovery of javamide-I/javamide-II) and separately (for the quantification of javamide-I/javamide-II). For the NMR confirmation, the equally mixed green coffee extract sample was extracted with 70% methanol (50 ml per 1 g green coffee) with gentle shaking at room temperatures for 24 hr. Then, the samples were extracted twice, providing 1st and 2nd extraction samples. The 1st and 2nd extraction samples were utilized to isolate javamide-I/javamide-II for NMR analysis and determine the extraction recovery. Meanwhile, for the quantification of javamide-I/javamide-II in each green coffee extract sample, seven green coffee samples were individually extracted using the same extraction method.

### 2.4. NMR Analysis

The peaks of javamide-I/javamide-II were isolated from the green coffee extract using the method described previously. The chemical structures of isolated javamide-I/javamide-II were verified using NMR spectroscopic methods. For NMR experiments, the samples were prepared by dissolving javamide-I/javamide-II (20 mg) in d6-DMSO (0.75 mL). ^1^H and ^13^C spectra were acquired at ambient temperature on the JEOL BCX-400 NMR spectrometer operating 400 MHz for ^1^H and 100 MHz for ^13^C.

### 2.5. Quantification of Javamide-I/Javamide-II in Green Coffee Extract

Seven green coffee extract samples were individually analyzed using the HPLC method as described previously. Since the extraction was performed twice (1st and 2nd), the 1st and 2nd extraction samples were independently analyzed using the HPLC method to determine the amounts of javamide-I/javamide-II in each extraction. The amounts of javamide-I/javamide-II were quantified using CoulArray electrochemical detector with four electrode channels (ESA, Chelmsford, MA).

### 2.6. Statistical Analysis

All statistical analyses were performed with the SigmaPlot 11.0 (Chicago, IL). Data points in all figures were represented as the mean ± SD of 5 samples.

## 3. Results and Discussion

### 3.1. HPLC Analysis of Javamide-I/Javamide-II Standards and Their Standard Curves

**Figure 1:**
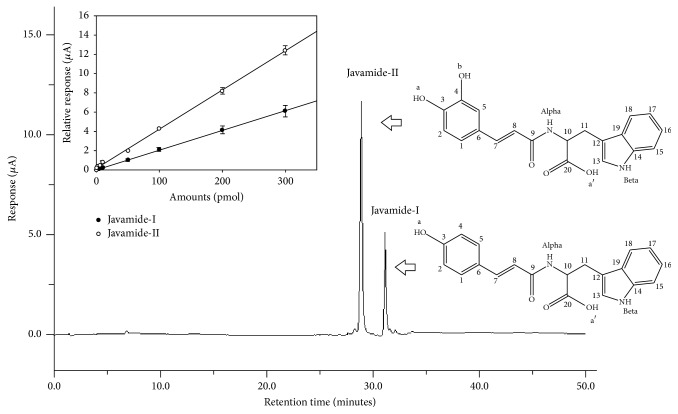
HPLC chromatograms and chemical structures of javamide-I/javamide-II. HPLC analysis was performed and the standard curve (insert; *f*(*x*) = 0.0204; *R*^2^ = 0.99 for javamide-I and *f*(*x*) = 0.0414; *R*^2^ = 0.99 for javamide-II) was produced, described in Materials and Methods. The peaks of the standards (*n* = 5) were detected using an electrochemical detector.

Javamide-I/javamide-II standards were separated and detected using the HPLC method as described in Materials and Methods (Figure 1). The HPLC condition was examined to improve the separation and resolution of the compounds using a gradient condition described in Materials and Methods. A HPLC condition was selected for optimizing the detection, separation, resolution, and reproducibility of the peaks of javamide-I/javamide-II in the chromatography. Total HPLC running time for the assay was 50 min, and javamide-I/javamide-II were detected at a retention time of 28.8 and 31.3 min, respectively. The peak areas of javamide standards (1, 10, 50, 100, 200, and 300 pmol) are utilized to produce their standard curves, which provided satisfactory linear responses at the concentrations between 1 and 300 pmol (for javamide-I correlation coefficient (*R*) = 0.99 (*f*(*x*) = 0.0204), and for javamide-II, correlation coefficient (*R*) = 0.99 (*f*(*x*) = 0.0414)). Also, the implemented limit of detection (LOD) and limit of quantification (LOQ) were 5/5.5 pmol, respectively (Figure 1).

### 3.2. Extraction and HPLC Analysis of Green Coffee Extract Mixture Sample

**Figure 2:**
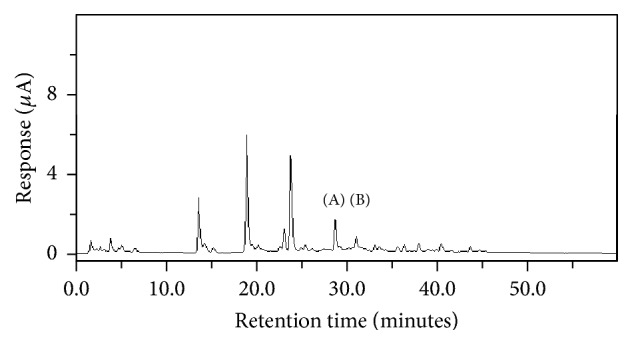
HPLC chromatograms of the extract prepared from the mixed green coffee sample. The mixture of seven green coffee samples (1 Nature Made Inc., 2 BioRise Nutrition, 3 GMX Naturals, 4 Natrogix, 5 Pro Nutrient Labs, 6 The Health Life, and 7 Wellsome Nutrition) was extracted with 70% MeOH (50 mL). The extract was analyzed using the HPLC method as described in Materials and Methods, and the peaks of javamide-II (A) and javamide-I (B) were detected using an electrochemical detector and confirmed by NMR spectroscopic methods.

Since all seven green coffee extract samples purchased from the market are fine powders, the sample was prepared by mixing equal amounts of the seven extract powders and was extracted with 70% methanol as described in Materials and Methods. After that, the HPLC analysis of javamide-I/javamide-II was performed as described previously. After several preparations of green coffee mixture samples, the 1 g green coffee per 50 mL 70% methanol was found optimal for extraction and HPLC separation of the javamide in the samples. The HPLC method provided excellent separation and resolution of the peaks of javamide-I/javamide-II in the green coffee samples (Figure 2). Like the chromatograms of javamide-I/javamide-II standards, javamide-I/javamide-II in the green coffee extract samples were detected at the same retention time (28.9 and 31.2 min).

### 3.3. NMR Confirmation of Javamide-I/Javamide-II

**Figure 3:**
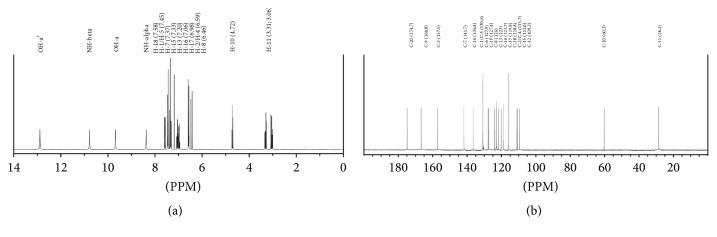
Proton and carbon NMR spectra for javamide-I. (a) Proton NMR spectrum and (b) carbon NMR spectrum. The proton and carbon numbers were depicted with chemical shifts in parenthesis.

**Figure 4:**
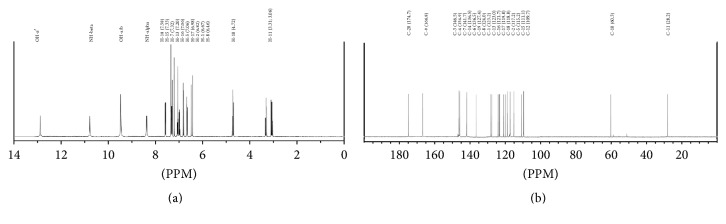
Proton and carbon NMR spectra for javamide-II. (a) Proton NMR spectrum and (b) carbon NMR spectrum. The proton and carbon numbers were depicted with chemical shifts in parenthesis.

For NMR confirmation, each peak of javamide-I/javamide-II was purified from the green coffee extract using the method described previously. The purified javamide-I was analyzed using NMR spectroscopic methods (Figures 3 and 4). The NMR data were following: 1H NMR (d6-DMSO, 400 MHz) *δ* 7.58 ((1H, d,* J*) 8.2 Hz, H-18), 7.45 ((1H, d,* J*) 8.2 Hz, H-1/H-5), 7.37 ((1H, d,* J*) 15.6 Hz, H-7), 7.33 ((1H, d,* J*) 7.8 Hz, H-15), 7.20 (1H, s, H-13), 7.06 ((1H, t,* J*) 7.3 Hz, H-16), 6.98 ((1H, t,* J*) 7.3 Hz, H-17), 6.59 ((1H, d,* J*) 8.2 Hz, H-2/H-4), 6.46 ((1H, d,* J*) 15.6 Hz, H-8), 4.72 ((1H, t,* J*) 7.3 Hz, H-10), 3.24 ((1H, dt,* J*) 6.0, 6.9 Hz, H-11), 9.68 (1H, br s, OH-a), 12.89 (1H, br s, OH-a′), 10.79 (1H, br s, NH-beta), 8.38 (1H, br s, NH-alpha); 13C NMR (d6-DMSO, 100 MHz) 174.7 (C, C-20), 166.8 (C, C-9), 157.5 (C, C-3), 141.7 (C, C-7), 136.4 (C, C-14), 130.6 (C, C-1), 130.6 (C, C-5), 127.9 (C, C-6), 127.4 (C, C-19), 124.0 (C, C-8), 123.0 (C, C-13), 121.7 (C, C-16), 119.8 (C, C-17), 118.8 (C, C-18), 115.7 (C, C-2), 115.7 (C, C-4), 111.5 (C, C-15), 109.3 (C, C-12), 60.3 (C, C-10), 28.2 (C, C-11). Based on the NMR data, the structure of the isolated compound was determined as being coumaroyl-L-tryptophan (*N*-coumaroyltryptophan; javamide-I). Likewise, the purified javamide-II was analyzed using NMR spectroscopic methods. The NMR data were following:

1H NMR (d6-DMSO, 400 MHz) *δ* 7.58 ((1H, d,* J*) 8.2 Hz, H-18), 7.33 ((1H, d,* J*) 7.8 Hz, H-15), 7.32 ((1H, d,* J*) 15.6 Hz, H-7), 7.20 (1H, s, H-13), 7.06 ((1H, t,* J*) 7.3 Hz, H-16), 7.06 (1H, s, H-5), 6.98 ((1H, t,* J*) 7.3 Hz, H-17), 6.82 ((1H, d,* J*) 8.7 Hz, H-2), 6.67 ((1H, dd,* J*) 8.2, 1.4 Hz, H-1), 6.46 ((1H, d,* J*) 15.6 Hz, H-8), 4.72 ((1H, t,* J*) 7.3 Hz, H-10), 3.24 ((1H, dt,* J*) 6.0, 6.9 Hz, H-11), 9.48 (1H, br s, OH-a, b), 12.89 (1H, br s, OH-a′), 10.79 (1H, br s, NH-beta), 8.38 (1H, br s, NH-alpha); 13C NMR (d6-DMSO, 100 MHz) 174.7 (C, C-20), 166.8 (C, C-9), 146.5 (C, C-3), 145.9 (C, C-4), 141.7 (C, C-7), 136.4 (C, C-14), 128.0 (C, C-6), 127.4 (C, C-19), 124.0 (C, C-8), 123.2 (C, C-1), 123.0 (C, C-13), 121.7 (C, C-16), 119.8 (C, C-17), 118.8 (C, C-18), 117.2 (C, C-2), 115.2 (C, C-5), 111.1 (C, C-15), 109.7 (C, C-12), 60.3 (C, C-10), 28.2 (C, C-11). Based on the NMR data, the structure of the isolated compound was determined as being (E)-(3-(3,4-dihydroxyphenyl)acryloyl)tryptophan (*N*-caffeoyltryptophan; javamide-II).

### 3.4. Extraction Recovery of Javamide-I/Javamide-II in Green Coffee Extract Samples

In order to determine the extraction recovery, the extraction was performed twice. The amounts of javamide-I in the 1st extraction were 95% higher than those of the 2nd extraction, high enough for no subsequent 3rd extraction (Table 1(a)). The recovery was determined by measuring the amounts following the addition of javamide-I standard (0.2 mg) and found to be more than 98% (Table 1(b)). Likewise, the amounts of javamide-II in the 1st extraction were 96% higher than those of the 2nd extraction (Table 2(a)) and the recovery was found to be more than 98% (Table 2(b)).

### 3.5. HPLC Quantification of Javamide-I/Javamide-II

For the quantification of javamide-I/javamide-II, seven green coffee extract samples were individually extracted and prepared for HPLC analyses using the same extraction method described in Materials and Methods. Javamide-I was detected in five samples at the range of 0.15–0.52 mg per g (Table 3). However, javamide-II was detected in all seven green coffee samples between 0.28 and 2.96 mg per g (Table 4), suggesting that javamide-II could be present in higher amounts than javamide-I in the green coffee extract samples, which was also confirmed in roasted coffee beans (data not shown here). Altogether, this study clearly suggests that javamide-I/javamide-II can be found in green coffee extract products sold in the market, but their amounts are likely to be significantly different depending on green coffee extract brands.

## 4. Conclusion

In this paper, javamide-I/javamide-II were extracted from seven green coffee extract products sold in the market and their amounts were quantified using the HPLC method which provided accurate and reliable measurement of javamide-I/javamide-II with excellent peak resolution and low detection limit. Javamide-II was detected in all seven green coffee extract samples at the range between 0.28 and 2.96 mg per g green coffee, but javamide-I was detected only in five samples at the range of 0.15–0.52 mg per g green coffee, indicating that the amount of javamide-II may be found higher than that of javamide-I in green coffee extract products. This study suggests that different amounts of javamide-I/javamide-II can be found in green coffee extract products and their amounts are likely to be very different depending on green coffee extract brands.

## Figures and Tables

**(a):** 

Sample	1st (mg)	2nd (mg)	1st and 2nd (mg)	1st/2nd (%)
S1–S7	0.20 ± 0.01	0.01 ± 0.001	0.21 ± 0.02	95.0

**(b):** 

Sample	Initial (mg)	J-I^added^ (mg)	1st (mg)	2nd (mg)	Yield (%)
S1–S7	0.2	0.2	0.39 ± 0.03	0.02 ± 0.002	98.0

**(a):** 

Sample	1st (mg)	2nd (mg)	1st and 2nd (mg)	1st/2nd (%)
*S1*–*7*	1.01 ± 0.02	0.03 ± 0.001	0.63 ± 0.02	97.1

**(b):** 

Sample	Initial (mg)	J-II^added^ (mg)	1st (mg)	2nd (mg)	Yield (%)
S1–7	1	1	1.89 ± 0.05	0.07 ± 0.005	98.0

**Table 3:** The amounts of javamide-I in seven green coffee samples. Seven green coffee extract samples (S1–S7) as described in Table 1 were individually extracted twice with 70% MeOH extracts (50 mL), providing the 1st and 2nd extracts. The amounts of javamide-I were presented as mg per 1 g green coffee sample (*n* = 5).

Sample	1st (mg)	2nd (mg)	1st and 2nd (mg)	1st/2nd ratio (%)
S1	0.41 ± 0.02	0.01 ± 0.001	0.42 ± 0.02	97.0
*S2*	0.51 ± 0.02	0.01 ± 0.001	0.52 ± 0.02	97.6
*S3*	0.15 ± 0.01	ND	0.15 ± 0.01	—
*S4*	ND	ND	—	—
*S5*	0.15 ± 0.01	ND	0.15 ± 0.01	—
*S6*	ND	ND	—	—
*S7*	0.17 ± 0.02	ND	0.17 ± 0.02	—

**(a):** 

Sample	1st (mg)	2nd (mg)	1st and 2nd (mg)	1st/2nd ratio (%)
S1	1.68 ± 0.05	0.07 ± 0.005	1.75 ± 0.05	95.6
S2	2.88 ± 0.06	0.08 ± 0.005	2.96 ± 0.06	97.0
S3	0.68 ± 0.02	0.02 ± 0.001	0.70 ± 0.02	96.4
S4	0.27 ± 0.01	0.01 ± 0.001	0.28 ± 0.01	95.2
S5	0.65 ± 0.02	0.02 ± 0.001	0.67 ± 0.02	96.2
S6	0.27 ± 0.01	0.01 ± 0.001	0.28 ± 0.01	95.2
S7	0.76 ± 0.02	0.02 ± 0.001	0.78 ± 0.02	96.8

**(b):** 

Sample	Javamide-I (mg)	Javamide-II (mg)
S1	0.42 ± 0.02	1.75 ± 0.05
S2	0.52 ± 0.02	2.96 ± 0.06
S3	0.15 ± 0.01	0.70 ± 0.02
S4	ND	0.28 ± 0.01
S5	0.15 ± 0.01	0.67 ± 0.02
S6	ND	0.28 ± 0.01
S7	0.17 ± 0.02	0.78 ± 0.02
